# Nutritional Composition of Edible Insects Consumed in Africa: A Systematic Review

**DOI:** 10.3390/nu12092786

**Published:** 2020-09-11

**Authors:** Zabentungwa T. Hlongwane, Rob Slotow, Thinandavha C. Munyai

**Affiliations:** School of Life Sciences, University of KwaZulu-Natal, Private Bag X01, Scottsville 3209, South Africa; slotow@ukzn.ac.za (R.S.); munyaic2@ukzn.ac.za (T.C.M.)

**Keywords:** entomophagy, Africa, edible insects, nutrition, food security

## Abstract

Edible insects are an important protein rich natural resource that can contribute to resilient food security. Edible insects not only play an important role in traditional diets, but are also an excellent source of protein in traditional dishes in Africa. We systematically searched Web-of-Science and Google Scholar from year 2000–2019 for studies on the consumption of insects and their nutritional composition in Africa, resulting in 98 eligible papers, listing 212 edible insect species from eight orders. These insects were rich in protein, fats, and fibre. The highest protein content was reported for Lepidoptera (range: 20–80%). Coleoptera had the highest carbohydrate content (7–54%), while Lepidoptera had the highest fat content (10–50%). Considering the excellent source of nutrition, and potential socio-economic benefits, from edible insects, they can contribute strongly to improved food security, and rural development in developing countries. In addition, edible insects can be used as a sustainable food source to combat food shortages in the future, for example, providing resilience during times of drought or other climate stressors.

## 1. Introduction

Consumption of insects has recently received more attention because of their promising potential for contributing to livelihoods and mitigating food security problems around the world [1,2,3]. Food security problems are caused by an enormous increase in the global human population, which is estimated to increase to approximately 9 billion people by 2050 [1], resulting in a 70% increase in food demand, and an increase in food prices [1,4,5]. The increase in food prices will prompt the search for cheap alternative sustainable protein sources [1]. Entomophagy, which refers to the consumption of insects by humans, is an environmentally friendly approach to increasing food for consumption, and contributing to food security across the world [2,5,6,7].

Edible insects might be a solution to food shortages, owing to their promising potential in contributing to livelihoods and mitigating food security problems around the world [1,2,3]. Insects are consumed as food in Thailand [8,9], China [10,11], Mexico [12,13,14,15], Latin America [16], Japan [17], and Africa [18]. According to van Huis [1], approximately 2 billion people worldwide regularly consume insects as part of their diets. The consumption of insects is not a new phenomenon, as it dates back to before the development of agriculture when humans relied on gathering plants and hunting wild animals [4,11,19]. 

Edible insects have played a very important traditional role in nutritious diets in various countries in Africa [18,20]. In addition, edible insects are an important natural resource that is used as a coping strategy, particularly in months of food shortage [21,22,23]. Unfavourable climatic conditions experienced in Africa affect small scale animal husbandry and reduce animal protein production, so diets are then supplemented with edible insect protein [22]. Edible insects provide significant socio-economic and ecological benefits for developing countries [24,25]. Approximately 500 species of edible insects are consumed in Africa and form part of traditional diets [18]. Of these 500 species, 256 species were consumed in the Central African region, 164 in southern Africa, 100 species in eastern Africa, 91 in western Africa, and only eight species in northern Africa [18]. Insects are consumed among different African cultures because of their taste, cultural importance, and nutritive value, and as a supplementary food when staple food is limited [1,3,25,26,27].

Various studies in Africa have focused on studying the nutritional content of a single species, group, or genus [28,29,30,31,32]. Little is known about the diversity and nutritional content of various insects consumed in Africa. Therefore, the current study will review the existing literature on the diversity of insects, and their nutritional status in Africa, and, therefore, compile information on the nutrient composition of edible insects consumed in Africa. This will be done by asking the following questions: (1) What is the nutritional value of edible insects consumed in Africa, (2) what are the most consumed, and (3) the most studied insect species, in terms of nutrition, in Africa?

## 2. Materials and Methods 

### 2.1. Search Strategy

To explore the diversity and nutritional status of edible insects in Africa, we followed the PRISMA guidelines for a systematic review. Peer-reviewed literature was obtained using the Thomson Reuters’ Web of Science database (https://apps.webofknowledge.com) and google scholar (https://scholar.google.co.za/) looking for publications that researched entomophagy in Africa, edible insects, diversity, nutrient content of edible insects, and consumption of insects. To source information, the following key words and phrases were used, “entomophagy”, “edible insects”, “diversity of edible insects”, “entomophagy in Africa”, “edible insects in eastern Africa”, “edible insects in north Africa”, “edible insects in western Africa”, “edible insects in Central Africa”, “edible insects in southern Africa” and “nutrient content of edible insects”. We also screened references included in selected articles in order to identify studies that might be relevant but did not appear in our search. We limited the search to literature published from 2000 to 2019. We started in the year 2000 because it was a starting point where most researchers began investigating the use of edible insects as a food source and as a solution to combat food insecurity problems [33,34].

### 2.2. Data Collection 

Data from the selected articles were independently screened and extracted by a single author (Z.T.H). The search result was done by reading the title and abstract of the retrieved papers to determine if the article was relevant to the study. Once it was determined that the article was relevant, the full text of the selected articles was further analysed to extract relevant information. The information that was collected and extracted after full text reading from each article included year, study area and country, study insect species, reported nutrient composition of insects, consumption stage of an insect, main research findings, and conclusions. Collected articles were categorised by country and insect order.

### 2.3. Inclusion and Exclusion Criteria

#### 2.3.1. Inclusion Criteria

- Original research articles and review papers focusing on entomophagy, nutrient composition of single or multiple edible insect species.
- Articles published in English.
- Articles of work done in African countries.
- Articles that reported nutrient composition of edible insects.

#### 2.3.2. Exclusion Criteria

- Conference papers, editorial material, book chapters
- Articles on insect rearing and farming.

### 2.4. Data Quality

To evaluate the quality of studies included in this systematic review, we assessed quality based on the following criteria: (1) A clear food description (scientific name(s) of insects studied or genus), (2) a clear description on the part of the insects used for analysis, e.g., whole, head, abdomen, indication of geographic origin of the insects, and the country where it is used as food in Africa, (3) analytical method used, number of analytical samples, (4) clear indication of whether the nutritional composition was based on the dry weight. Studies were included if they meet all the above criteria.

### 2.5. Data Analysis

The methods and data sources used in the included studies were highly heterogeneous and a statistical meta-analysis was not possible. Instead, a more narrative synthesis approach was used, and data from each study were tabulated. We synthesised the results according to study species and mean values of all insect species belonging to the same insect order were calculated and represented in bold, the nutritional composition of consumed species were presented in the table, most consumed species in different countries were presented graphically. 

## 3. Results and Discussion

**Figure 1:**
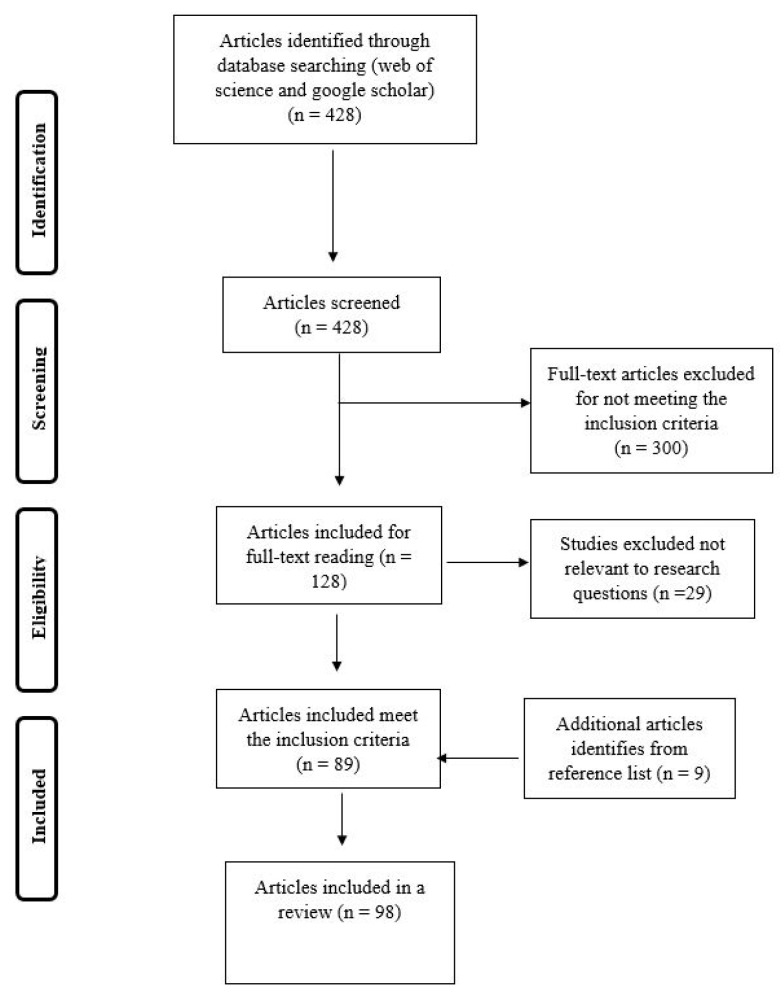
Flow chart of the study selection process for systematic review of the nutritional composition of edible insects.

A total of 428 papers were identified for potential inclusion; after checking the title and abstract, 300 articles were excluded because they did not meet the inclusion criteria. From here, 128 articles were selected for full-text reading; from these, 29 articles were further excluded because they were not relevant or not conducted in Africa. After reading the full-text, 89 studies met all inclusion criteria, and a further nine articles were identified through screening references and confirming inclusion criteria were met. In total 98 articles were included in a systematic review (Figure 1). 

### 3.1. Consumption of Insect Patterns in Africa

**Figure 2:**
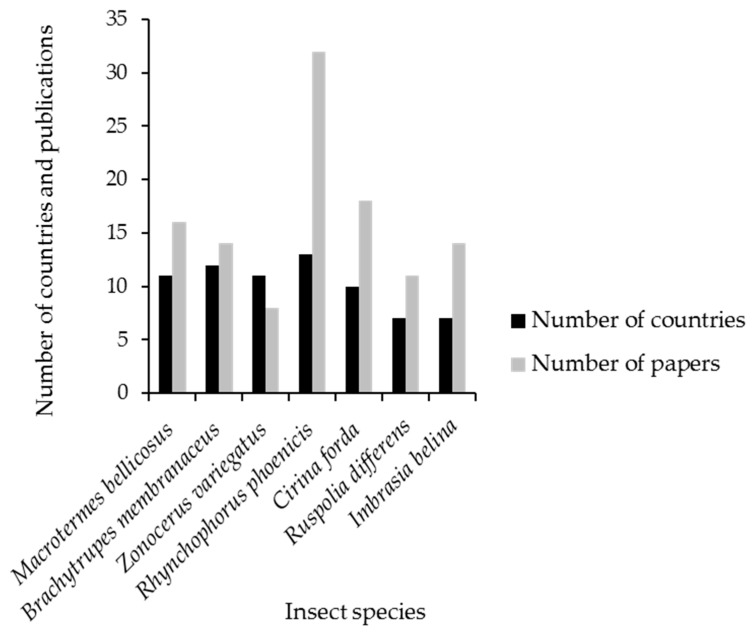
The number of countries with journal peer-reviewed articles published on the most consumed and economically important insects in Africa.

For the research articles published since 2000, a total of 212 edible insect species from nine orders were recorded and are potentially consumed in different African countries (Appendix A). Of these, 41% were Lepidoptera, 23% Orthoptera, 15% Coleoptera, 12% Blattodea (including both cockroaches and termites as recently classified), 4% Hemiptera, and Hymenoptera, Diptera, Blattodea, and Mantodea each contributed <1%. Rhynchophorus phoenicis (African palm weevil) and Cirina forda (Pallid emperor moth) were the most studied species in Africa, with 32 publications from 12 countries, and 18 publications from 10 countries, respectively (Figure 2). Most research has been done in the western African countries, particularly in Nigeria, mainly on Rhynchophorus phoenicis and Cirina forda, which are the most consumed species in West Africa. However, southern African countries (Zimbabwe, South Africa, and Bostwana) have the highest number of consumed species, but little research has been done on nutritional content and consumption patterns of edible insects.

### 3.2. Nutrient Composition of Edible Insects

A compilation of nutrient composition of 54 edible insects based on the dry matter is presented in Table 1. Percentage of fat, protein, moisture, and ash content were calculated based on dry weight of the insect when ready for preparation to eat, noting that, in some cases, the insects had been processed since collecting. The highest protein was reported in Lepidoptera (range: 12–79%) and Orthoptera (12–73%), while the lowest protein content ranging from (0–39%) was reported for Blattodea.

The crude fibre was reported to be higher in Coleoptera (2–28%) and Lepidoptera (2–16%), while the crude fibre content was reported to be lowest in Hemiptera (0–5%). Lepidoptera had the highest moisture content (3–86%), while Blattodea had the lowest moisture content (2.8–3%) (Table 1).

The highest carbohydrate content was recorded in Coleoptera (13–52%) and Orthoptera (15–47%), while the lowest carbohydrate content was recorded in Blattodea (0–32%). Fat content was the highest in Lepidoptera (2–55%) and lowest in Orthoptera (2–16%) (Table 1).

Orthoptera had the highest iron content (0.3–910 mg/100 g) followed by Blattodea (27–332 mg/100 g), while Hemiptera had the lowest iron content (0–20 mg/100 g). Calcium content was higher in Blattodea (18–132 mg/100 g) and lowest in Lepidoptera (8–15 mg/100 g). The highest Phosphorus was recorded in Lepidoptera (100–730 mg/100 g) and the lowest in Orthoptera (106–125 mg/100 g). Magnesium content was the highest in order Lepidoptera (1–160 mg/100 g), while Blattodea had the lowest magnesium content (0.1–0.3 mg/100 g) (Table 1).

Edible insects are widely consumed in Africa, and play an important role in nutritious diets. However, the preference and consumption of insects vary with species and orders. Lepidoptera caterpillars were the most consumed order, and they are the most preferred species because of their nutritional value, they are rich in protein, fats, and essential micronutrients [6,54]. In addition, several caterpillar species play an important role in income generation in rural areas in southern Africa, Uganda, and Nigeria [18,22,55]. 

Studies from western and Central Africa indicated that *Rhynchophorus phoenic* (palm weevil), and *Cirina forda* (pallid emperor moth) were the commonly consumed species [18,24,56]. The palm weevil and pallid emperor moth are a delicacy in western and Central Africa, and, in addition, these species were of economic importance in Nigeria, Cameroon, Benin, and Ghana [57]. In southern Africa, the literature indicates that the most consumed or preferred species were *Imbrasia belina* (mopane worm), *Macrotermes natalensis*, *falciger*, and *bellicosus* (termites) [28,50,58]. While in eastern Africa, the most consumed species were *Ruspolia nitidula* and *differens* (grasshoppers), [22,59,60,61]. Mopane worms, and termites are an important part of food culture in different ethnic groups in southern Africa [18,59]. Moreover, the trade of mopane worms and termites plays an important role in rural food security and income generation, as it provides rural people with household income [28,50,57,58]. 

Edible insects are a good source of protein content, which ranges from 12–79% of dry matter, which is consistent with studies from China, Germany, and Asia [6,10]. The protein content reported in edible insects is higher than protein found in chicken (43%) or beef (54%) [28,62]. The high protein content found in edible insects could help to combat protein deficiency in Africa. Protein deficiency is a major contributor to human malnutrition [63], and, in Africa, protein deficiency is the most common form of malnutrition, which needs to be addressed to halt starvation [64]. Therefore, including edible insects in daily diets might help reduce malnutrition rates. 

Moisture content ranged from 1–7.5%, which is relatively low, such that most edible insects have longer preservation periods, and the risk of microbial deterioration and spoilage is minimal [29,42,65]. Unlike beef or chicken, which are prone to decay (unless refrigerated), edible insects can be stored for longer periods, especially during the dry season when food shortage is higher [42]. However, three caterpillars (*Gonimbrasia* (Nudaurelia) *alopia*, *Anaphe panda*, and *Pseudontheraea discrepans*) had higher moisture (>60%), meaning they are prone to spoilage and their preservation period is shorter unless processed in some manner. Siulapwa et al. [29] reported similar results, where caterpillars *Imbrasia belina* and *Gynanisa maja* had higher moisture content than other species. To increase shelf life, caterpillars are usually degutted, washed in boiling salt water, or roasted before drying in the sun, then packed in large sacks and containers [23,66]. 

Edible insects contain fat content ranging from 1–67%. The fat content of edible insects are higher in the larval stage. For example, a palm weevil, which is a beetle larva that is consumed as a delicacy in western Africa, contained the highest fat content of 67%. These results are consistent with Bukkens [67], who reported that Lepidopteran caterpillars and palm weevil larvae contain higher fat than any other insect species. Edible insects can be used to provide essential fatty acids required by the human body [10,68]. In addition, fat plays an important role in providing the human body with energy, which means that consuming insects such as *Rhynchophorus phoenicis*, *Imbrasia belina*, *Anaphe panda*, and *Brachytrupes membranaceus*, may help provide people with energy, thereby reducing malnutrition associated with energy deficiencies in developing countries [4,10,69].

Carbohydrates play a very important role in human nutrition as they are the primary source of energy. Carbohydrates found in edible insects varied from 5–51% [19,70]. Therefore, edible insects can be used as a source of carbohydrates, as they contain relatively high amounts of polysaccharides, which play an important role in enhancing the immune system of the human body [10]. In addition, carbohydrates are an essential nutritive element in the human body [29]. Species such as *Oryctes monoceros* and *Gryllotalpa africana*, reported in the current study, contained a high amount of carbohydrates; therefore, edible insects can be included in human diets to provide a good source of carbohydrates [29].

Excellent source of iron and zinc found in some edible insects indicate that edible insects could be used to combat malnutrition deficiencies such as zinc and iron deficiency anemia, which is prevalent in Africa [37]. Species such as *Zonocerus variegatus*, *Pseudacathotermes spinige*, and *Macrotermes herus* contained high iron content of 910, 332, and 161 mg/100 g respectively, which means that these species can be used as a good source of Iron. Zinc content was notably high in insects such as *Zonocerous variegatus* (29 mg/100 g) and *Rhyncophorus phoenicis* (26.5 mg/100 g) the Zinc content found in these insects exceed the daily recommended intake of 3.0–14 mg/100 g. Rumpold and Schluter [6] reported that Iron and Zinc content found in edible insects is generally higher than the Zinc and Iron content found in pork, beef, or chicken; therefore, edible insects might be a solution in fighting Iron and Zinc deficiency. Zinc and Iron deficiency are one of the health problems faced by many women of reproductive age and children in developing countries [37]. Therefore, consumption of edible insects might provide a solution to Iron deficiency health problems, such as anemia, reduced physical activity, and maternal mortality [37,71].

Edible insects reported in the current study contained a low amount of Vitamin A, B2, and C. The 100 g dry matter of edible insects reported in this study did not contain enough daily recommended Vitamin A (500–600 mg) or C (45 mg). As such, Chen et al. [10] reported that to meet the daily recommended amount of Vitamin C, insect tea derived from the excrement of insects is an option. This tea contains up to 15.04 mg of Vitamin C per 100 g, and the consumption of 300 mL of insect tea per day makes 45 mg of Vitamin C, which is the daily recommended amount of vitamin C for adults [10]. Contrary to findings reported in this study, Bukkens [67] reported that Vitamin B1, B2, and B3 content found in an edible house fly is richer than the Vitamin B1, B2, and B3 found in chicken, beef, or salmon. In addition, edible crickets contain twice more Vitamin B12 than the beef [69]. Igwe et al. [72] found that *Microtermes nigeriensis* contain a favourable high source of Niacin, Thiamine, Vitamin A, and C. Vitamins play an important role in human nutrition, as Vitamin C is important for human growth, development, and repair of various body tissues [73]. The excellent source of Vitamins found in some edible insects shows that insects have a great potential of being used as a healthy food supplement for malnourished people, or to prevent malnutrition [24]. 

There were several limitations to this review, which included studies reported in English only and excluded studies published in other languages used in Africa. There were significant gaps in data available on the nutritional composition of edible insects consumed in Africa. Most publications focused on a single macronutrient content, especially protein, carbohydrates, fats and fibre, and other nutrients, especially minerals, are not included in analyses. In addition, research focused on reporting the nutritional composition of economically important species such as *Imbrasia belina*, *Macrotermes natalensis*, *bellicosus* and *falciger, Rhynchophorus phoenics*, and *Cirina forda*. Strengths of this review incudes the robust approach to combine the nutritional composition of consumed insects in Africa, previous studies have focused on documenting the nutritional composition of single, or a group of, insects that are consumed in Africa.

This review reported combined nutritional data of consumed insects in Africa; this information can be useful to policy makers in the health and nutrition sector by including insects in food and nutrition policies. Health officials need to motivate people to include insects in their daily diets, particularly the most vulnerable groups such as elderly people, women, and children, with the aim to improve the quality of life for people. In addition, farming and rearing of insects by the agricultural sector need to be adopted to ensure that insects are easily accessible and available all year even when they are out of season in nature. Insects can be included as an ingredient in other food products such as bread, maize powder, chocolate, and biscuits to overcome discomfort and fear associated with eating whole insects in some groups of people. Future studies are required to research sustainable ways of farming and rearing insects in Africa and the implication that might have on the environment.

## 4. Conclusions

Meeting global food demand and halting poverty in Africa are among the greatest challenges, and these challenges are expected to continue if sustainable and innovative measures are not put into place. In 2017, approximately 256 million people were reported to be undernourished in Africa [74]. There is no doubt that Africa is far from achieving Sustainable Development Goal 2, which is to end hunger, achieve food security and improved nutrition, and promote sustainable agriculture by 2030. Edible insects are widely consumed in Africa, and they play an important socio-economic role for rural communities in Africa, by providing nutritious diets (this review), and income opportunities to traders and harvesters [22,75,76]. In addition, edible insects are a traditional delicacy, and are used as an emergency food source during times of food shortage [57]. They are rich in protein, carbohydrates, amino acids, and micronutrients such as Zinc and Iron. This implies that edible insects have a potential of contributing in sustainable diets, while assuring food security, and improving livelihoods of African people.

## Figures and Tables

**Table 1:** Nutritional composition of edible insects, based on dry matter, from six orders consumed by people in Africa.

Scientific Name	Stage of Consumption	Protein (%)	Crude Fibre (%)	Moisture (%)	Ash (%)	Carb (%)	Vitamin A (mg/100 g)	Vitamin B2 (mg/100 g)	Vitamin C (mg/100 g)	Fe (mg/100 g)	Ca (mg/100 g)	Zn (mg/100 g)	P (mg/100 g)	Mg (mg/100 g)	Fats (mg/100 g)	Reference
**Blattodea (termites and cockroaches)**		**33.2 ± 14.5**	**4.7 ± 3.9**	**2.9 ± 0.1**	**5.2 ± 2.5**	**23.2 ± 0**	**2.7 ± 0.2**	**1.8 ± 0.2**	**3.2 ± 0.2**	**86 ± 96.8**	**54.1 ± 42.6**	**13.8 ± 3.5**	**125 ± 11**	**0.2 ± 0.1**	**22.2 ± 9.8**	
*Periplaneta Americana*	Adult	39.6	13.1		6.2											[35]
*Macrotermes nigeriensis*	Adult	35.9	5.5		5.8											[35]
*Macrotermes bellicosus*	Adult	20.4	2.7	2.8	11.3	23.2	2.9	2.0	3.4	27.0	21.0		136.0	0.2	36.1	[36]
*Macrotermes natalensis*	Adult	22.1	2.2	3.0	4.1		2.6	1.5	3.0	29.0	18.0		114.0	0.3	21.4	[36]
*Pseudacathotermes spinige*	Adult				6.8					332.0	84.7	11.9				[37]
*Macrotermes spp.*	Adult				2.4					93.9	83.7	8.1				[37]
*Macrotermes herus*	Adult				6.8					161.0	132.0	14.3				[37]
*Macrotermes bellicosus*	Adult	40.7			5.7					42.7		16.9			8.4	[38]
*Macrotermes bellicosus*	Adult	20.4	2.7	2.8	2.9		2.9	2.0	3.4	27.0	21.0		136.0	0.2		[36]
*Syntermes soldiers*	Adult	64.7			4.2					32.5		17.6			23.0	[38]
*Macrotermes natalensis*	Adult	22.1	2.2	3.0	1.9		2.6	1.5	3.0	29.0	18.0		114.0	0.3		[36]
**Coleoptera (beetles)**		**32.8 ± 11.5**	**6.2 ± 7.8**	**7.6 ± 15.7**	**4.7 ± 2.7**	**22.6 ± 13.2**	**11.2 ± 1.4**	**1.9 ± 0.9**	**5.4 ± 1.2**	**14.1 ± 8.9**	**43.6 ± 14.3**	**14.4 ± 12.1**	**109.6 ± 48.5**	**10.1 ± 4.2**	**29.1 ± 16.6**	
*Analeptes trifasciata*	Larvae	20.1	2.0	2.2	5.1		12.5	2.6	5.4	18.2	61.2		136.4	18.2		[36]
*Oryctes boas*	Larvae	26.0	1.5	1.9	1.5							2.3				[6,36]
*Oryctes monoceros*	Larvae	26.4		4.7	7.8	51.6										[39]
*Aphodius rufipes*	Larvae	22.4	28.1	3.3	2.7	13.1				30.9	42.2			11.7	30.5	[36]
*Rhynchophorus phoenicis*	Larvae	28.4	2.8	2.7	2.7		11.3	2.2	4.3	12.2	39.6	26.5	126.4	7.5	66.6	[6]
*Oryctes rhinoceros*	Larvae	50.5								4.5					38.1	[6]
*Oryctes owariensis*	Larvae	50.6		8.4	7.7	14.3									18.9	[40]
*Eulopida mashona*	Larvae	46.3	14.8		10.9	16.2									11.8	[41]
*Heteroligus meles*	Larvae	38.1	3.0	1.0	5.8	20.1									32.0	[42]
*Rhynchophorus phoenicis*	Larvae	50.0	2.6	1.2	4.9	20.2									21.1	[42]
*Rhynchophorus phoenicis*	Larvae	28.4	2.8	2.7	2.7		11.3	2.2	4.3	12.2	39.6		126.4	7.5		[36]
*Analeptes trifasciata*	Larvae	29.6	2.0	2.2	4.2		12.5	2.6	5.4	18.2	61.3		136.4	6.1		[36]
*Oryctes boas*	Larvae	26.0	3.4	1.9	1.5		8.6	0.1	7.6	2.3	45.7		130.2	6.3		[36]
*Apomecyna parumpunctata*	Larvae	16.8	5.4	59.4	3.0						15.7		1.5	13.5	13.9	[43]
**Hemiptera (bugs)**		**39.3 ± 4.0**	**5.3 ± 0**	**4.9 ± 0**	**1.7 ± 0**	**6.3 ± 1.3**	**0.2 ± 0**	**0.9 ± 0**		**20.2 ± 0**	**91.0 ± 0**	**46.0 ± 0**	**57 ± 0**	**109 ± 0**		
*Encosternum delegorguei*		43.3	5.3	4.9	1.7	5.0	0.2	0.9		20.2	91.0		575.0	109.0	45.0	[6]
*Encosternum delegorguei*		35.2		4.9	1.7	7.6				20.2	91.0	46.0		109.0		[28]
**Hymenoptera (bees and ants)**		**33.9 ± 9.2**	**7.7 ± 4.6**	**3.9 ± 0.1**	**4.1 ± 3.2**		**12.4 ± 0**	**3.2 ± 0**	**10.3 ± 0**	**17.8 ± 6.6**	**21.6 ± 6.3**	**7.5 ± 2.5**	**115.6 ± 9.6**	**7.8 ± 2.6**	**42.9 ± 4.7**	
*Apis mellifera*	Adult	21.0	2.0	3.8	2.2		12.4	3.2	10.3	25.2	15.4		125.5	5.2		[6,36]
*Carebara vidua*	Adult	42.5	9.1		8,6					10.4	22.3	5.7	106.0	10.4	38.2	[6]
*Componotus spp.*	Adult	40.1	14.1		9.6											[35]
*Oecophylla longinoda*	Adult	37.8	12.3		7.3											[35]
*Crematogaster mimosa*	Adult				1.7					17.7	32.6	11.1				[37]
*Carebara vidua Smith*	Adult	40.8	6.9	3.9	1.6					10.7	22.2	5.7	106.0	10.4	47.5	[44]
*Apis mellifera*	Adult	21.0	2.0	3.8	2.2		12.4	3.2	10.3	25.2	15.4		125.0	5.2		[36]
**Lepidoptera (caterpillars)**		**46.3 ± 21.7**	**5.9 ± 5.4**	**29.3 ± 36.5**	**4.6 ± 2.2**	**18.0 ± 13.0**	**3.1 ± 0.2**	**1.7 ± 0.6**	**2.8 ± 1.0**	**15.4 ± 22.2**	**9.4 ± 2.3**	**10.6 ± 2.2**	**320.7 ± 367.9**	**18.9 ± 45.5**	**18.3 ± 14.8**	
*Anaphe venata*	Larvae	60.0	3.2	3.3			3.1	1.3	2.2	2.0	8.6		100.5	1.6		[6]
*Anaphe infracta*	Larvae	20.0	2.4	2.7			3.0	2.0	4.5	1.8	8.6		113.3	1.0		[6,36]
*Anaphe recticulata*	Larvae	23.0	3.1	3.2			3.4	2.0	2.2	2.2	10.5		102.4	2.6		[6,36]
*Cirina forda*	Larvae	20.2	1.8	4.4			3.0	2.2	2.0	64.0	15.4	8.6	110.0	1.9		[6,36]
*Imbrasia epimethea*	Larvae	73.1		79.8						13.0		11.1	402.0		12.4	[36]
*Imbrasia obscura*	Larvae	62.3		83.0											12.2	[45]
*Gonimbrasia* (*Nudaurelia*) *alopia*	Larvae	62.3		85.7											1.9	[45]
*Gonimbrasia* (*Nudaurelia*) *dione*	Larvae															[45]
*Pseudantheraea discrepans*	Larvae	48.9		72.2											21.3	[45]
*Anaphe panda*	Larvae	53.2		83.4											55.0	[6,33]
*Cirina butyrospermi*	Larvae	62.7	5.0		5.1					13.0						[46]
*Imbrasia belina*	Larvae	55.3	16.0		8.3	8.2				31.0		14.0	543.0	160.0		[6,47]
*Gynanisa maia*	Larvae	51.1	16.2		7.7	14.1									16.4	[47]
*Loba leopardina*	Larvae	25.8	14.7		6.6	40.2									12.6	[47]
*Imbrasia macrothyris*	Larvae	75.4														[33]
*Nudaurelia macrothyrus*	Larvae	75.4														[33]
*Gonimbrasia richelmanni*	Larvae	79.6														[33]
*Cirina spp.*	Larvae									64.0	7.0	8.6	1090.0	32.4		[48]
*Cirina butyrospermi*	Larvae	62.7			5.0								1160.0		14.3	[46]
*Hemijana variegata Rothschild,*	Larvae		8.3	5.9	5.2	9.5										[49]
*Anaphe infracta*	Larvae	20.0	2.4	2.7	1.6		3.0	2.0	4.5	1.8	8.6		111.3	1.0		[36]
*Anaphe recticulata*	Larvae	23.0	3.1	3.2	2.5		3.4	2.0	2.2	2.2	10.5		102.3	2.6		[36]
*Anaphe spp.*	Larvae	18.9	1.7	2.5	4.1		2.8	0.1	3.2	1.6	7.6		122.2	1.0		[36]
*Anaphe venata*	Larvae	25.7	2.3	3.3	3.2		3.1	1.3	2.2	2.0	8.6		100.5	1.6		[36]
**Orthoptera (grasshoppers, locust and crickets)**		**39.8 ± 21.1**	**6.4 ± 4.8**	**3.5 ± 1.7**	**5.5 ± 4.0**	**26.8 ± 14.5**	**3.0 ± 3.5**	**0.2 ± 0.4**	**2.9 ± 4.0**	**120.1 ± 298.8**	**17.3 ± 15.8**	**91.1 ± 99.8**	**119.7 ± 12.7**	**2.8 ± 3.8**	**20.8 ± 18.9**	
*Brachytrupes membranaceus*	Adult	53.4	15.0	3.4	6.0	15.1	0.0	0.0	0.0	0.7	9.2		126.9	0.1	53.0	[6,47]
*Cytacanthacris naeruginosus unicolor*	Adult	12.1	2.1	2.6			1.0	0.1	1.0	0.4	4.4		100.2	0.1		[6,36]
*Zonocerus variegatus*	Adult	26.8	2.4	2.6			6.8	0.1	8.6	910.0	42.2		131.2	8.2		[6,36]
*Gryllotalpa africana*	Adult	22.0	7.5		12.6	47.2									10.8	[47]
*Henicus whellani*	Adult	53.6	10.6		14.0										4.3	[50]
*Cartarrtopsilus taeniolatus*	Adult	40.6	13.3		6.9											[35]
*Zulua cyanoptera*	Adult	33.7	13.3		6.6											[51]
*Ornithacris turbida*	Adult	42.7	2.0		4.5	18.2									2.0	[47]
*Ruspolia differens*	Adult	72.7	6.3		4.6			1.2	0.1	13.0	24.5	12.4	121.0	33,1	46.2	[6]
*Anacridium melanorhodon melanorhodon* (Walker)	Adult	66.2	8.4	7.5											12.4	[52]
*Zonocerous variegatus*	Adult	62.7	3.6		1.2		8.9	0.1	9.8		2.0	29.0				[6]
*Brachytrypes membranaceus* L	Adult															[53]
*Zonocerous variegatus*	Adult	26.8	2.4	2.6	1.2					2.0	42.2		131.2	8.2		[36]
*Brachytrupes* spp.	Adult	65.4			4.9					33.6		232.0			16.9	[38]
*Brachytrupes* spp.	Adult	6.3	1.0	3.4	1.8		0.0	0.0	0.0	0.7	9.2		126.9	0.1		[36]
*Cytacanthacris aeruginosus unicolor*	Adult	12.1	1.5	2.6	2.1		1.0	0.1	1.0	0.4	4.4		100.2	0.1		[36]
* Recommended daily intakes (mg/day) for adults										45.0	7.5–58.8	1300.0	3.0–14.0	700.0	220–260	[37]

Note the mineral abbreviations are Fe: Iron; Zn: Zinc; Ca: Calcium; P: Phosphorus; Mg: Magnesium. * Source [37]. Mean **±** standard deviation of insects belonging to the same insect order are highlighted in bold and species names are in italics.

## Appendix A

**Table A1:** Edible insects consumed in different African countries.

Order	Scientific Name/Morpho Species	Common Name	Country	Consumption Stage	References
Blattodea	*Periplaneta americana*	Common cockroach	Nigeria	Adult	[35]
Coleoptera	*Analeptes trifasciata*	Stem girdler	Nigeria	Larvae	[24,36,77]
Coleoptera	*Oryctes boas Fabr*	Rhinoceros beetle	Nigeria, Ivory Coast, Sierra Leone, Liberia, Democratic Republic of Congo, South Africa, Botswana, Namibia, Guinea Bissau	Larvae	[18,24,33,36,78,79]
Coleoptera	*Oryctes monoceros*	Rhinoceros beetle	Nigeria	Larvae	[24,33,36,39,56,79]
Coleoptera	*Aphodius rufipes*	Dung beetle	Nigeria	Larvae	[24,36,80]
Coleoptera	*Rhynchophorus phoenicis*	Palm weevil	Nigeria, Angola, Burkina Faso, Cameroon; Ghana, Cote D’ivioire, Democratic Republic of Congo, Liberia, Niger, Sao Tome, Togo, Benin, Guinea Bissau	Larvae, pupa and adult	[18,24,33,36,39,42,56,57,77,79,81,82,83,84,85,86,87,88,89,90,91,92,93,94,95,96,97,98]
Coleoptera	*Heteroligus meles*	Yam beetle	Nigeria	Larvae, pupa, adult	[24,36,42,77,79,91,99,100]
Coleoptera	*Eulepida mashona*	Beetle	Zimbabwe	Larvae/adult	[51,58]
Coleoptera	*Carbula marginella*	Beetle	Burkina Faso	Adult	[98]
Coleoptera	*oryctes sp.*	Beetle	Burkina Faso	Larvae	[98]
Coleoptera	*Oryctes rhinoceros* larva	Beetle	Nigeria; Cote D’ivoire	Larvae	[79,81,99,101,102]
Coleoptera	*Stenorcera orissa* Buq	Giant jewel beetle	Botswana, Zimbabwe	Winged adult	[58,78]
Coleoptera	*Eulepida anatine*	Beetle	Zimbabwe	Larvae	[58]
Coleoptera	*Eulepida nitidicollis*	Beetle	Zimbabwe	Larvae	[58]
Coleoptera	*Apomecyna parumpunctata*	African longhorned beetle	Nigeria	Larvae	[43]
Coleoptera	*Oryctes owariensis*	Beetle	Cote D’ivioire, Democratic Republic of Congo, South Africa, Angola, Malawi, Botswana, Mozambique, Zambia, Zimbabwe Nigeria, Ivory Coast, Sierra Leona, Guinea, Ghana, Equatorial Guinea, Guinea Bissau	Adult	[18,33,40]
Coleoptera	*Rhinoceros oryctes*	Beetle	Nigeria	Larvae, pupa, adult	[91]
Coleoptera	*Sitophilus oryzae*	Rice weevil	Nigeria	Larvae, pupa, adult	[91]
Coleoptera	*Callosobruchus maculatus*	Bean beetle	Nigeria	Larvae, pupa, adult	[91]
Coleoptera	*Dermestes maculatus*	Beetle	Nigeria	Larvae, pupa, adult	[91]
Coleoptera	*Cotinis nitida*	Beetle	Nigeria	Adult/larvae	[79]
Coleoptera	*Eulopida mashona*	Beetle	Zimbabwe	Adult/larvae	[47]
Coleoptera	*Sternocera funebris*	Beetle	Zimbabwe	Adult/larvae	[47]
Coleoptera	*Oryctes* spp Oliver	Beetle	Nigeria	Larvae	[77]
Coleoptera	*Augosoma centaurus*	Beetle	Cameroon	Adult, larvae	[57]
Coleoptera	*Phyllophaga nebulosa* (Harris)	Beetle larvae	Ghana	Larvae	[94,103]
Coleoptera	*Sitophilus zeamais*	Beetle	Ghana	Larvae, adult	[104]
Coleoptera	*Polycleis equestris*	Weevil	South Africa	Adult	[33]
Coleoptera	*Polycleis plumbeus*	Weevil	South Africa	Adult	[33]
Coleoptera	*Sipalus aloysii-sabaudiae*	Beetle	South Africa	Larvae	[33]
Coleoptera	*Teralobus flabellicornis*	Beetle	South Africa	Larvae	[33]
Coleoptera	*Sternocera orissa*	Beetle	South Africa	Larvae	[33]
Diptera	*Chaoborus edulis*		Malawi	Adult	[33]
Hemiptera	*Nezara viridula*	Southern green stink bug	Nigeria	Adult	[24,36,99]
Hemiptera	*Encosternum delegorgui* Spinola	Stink bug	South Africa, Zimbabwe, Swaziland, Malawi, Botswana, Namibia, Mozambique	Adult	[18,28,33,47,58,105]
Hemiptera	*Monomatapa insingnis* Distant	Cicada	Botswana	Adult	[78]
Hemiptera	*Aspongubus viduatus*	Melon bug	Sudan	Adult	[106]
Hemiptera	*Agonoscelis pubescens*	Sorghum bug	Sudan	Adult	[106]
Hemiptera	*Rhynchophorus spp.*	May bug	Nigeria, Cameroon	Larvae	[79,107]
Hemiptera	*Brevisana brevis*	African cicada	Zimbabwe	Ault	[47]
Hemiptera	*Ugada limbalis*	Cicada	Uganda		[108]
Hemiptera	*Pediculus capitata*		Angola, Malawi, South Africa, Zambia, Zimbabwe, Mozambique, Namibia, Botswana		[33]
Hymenoptera	*Apis mellifera*	Honey bee	Nigeria, Botswana, Cote D’ivioire, Cameroon, Zambia, Zimbabwe, Botswana, Angola, Mozambique, Tanzania, Senegal, Ghana, Lesotho, Benin, South Africa	Egg, larva, pupa	[2,18,33,36,38,77,78,91,107,109,110]
Hymenoptera	*Carebara vidua*	African thief ant	Botswana, Zimbabwe; Kenya Burundi, South Africa, Malawi, Zambia, Sudan, Namibia, Mozambique	Winged adult	[18,33,44,47,58,78,81,108]
Hymenoptera	*Plebeina hildebrandti* Friese	Stingless bee	Botswana	Adult	[78]
Hymenoptera	*Hypotrigona gribodoi* Magretti	Stingless bee	Botswana	Adult	[78]
Hymenoptera	*Cossus cossus*	Capenter ant	Cote D’ivioire	Adult	[102]
Hymenoptera	*Componotus spp.*	Ant	Nigeria	Adult	[35]
Hymenoptera	*Oecophylla longinoda*	African weaver ant	Nigeria, Cameroon	Adult	[35,93,107]
Hymenoptera	*Carebara lignata*	Ant	Zambia, South Africa, Democratic Republic of Congo, Zimbabwe, Botswana, Mozambique, Namibia, Sudan	Adult	[18]
Blattodea	*Macrotermes nigeriensis*	Termite	Nigeria	Winged adult, queen	[24,33,35,36,72,111,112]
Blattodea	*Macrotermes bellicosus*	Termite	Nigeria, Kenya, Uganda, Democratic Republic of Congo, Cameroon, Cote D’ivioire, Sao Tome, Togo, Liberia, Burundi, Ghana, Zimbabwe,	Winged adult, queen	[18,24,33,36,59,77,79,82,94,109,111,113,114,115]
Blattodea	*Macrotermes natalensis*	Termite	Nigeria, South Africa, Zimbabwe, Cameroon, Democratic Republic of Congo, Burundi, Malawi	Winged adult, queen	[24,33,36,47,58,75,99]
Blattodea	*Macrotermes falciger*	Termite	Democratic Republic of Congo; South Africa, Zimbabwe, Burundi, Zambia, Burkina Faso, Benin	Winged adult	[18,33,58,75,108,115,116,117]
Blattodea	*Macrotermes michaelseni*	Termite	South Africa	Winged adult	[75]
Blattodea	*Macrotermes subhyalinus*	Termite	Burkina Faso Zimbabwe, Cote D’ivioire, Rwanda, Uganda, Angola, Togo, Kenya	Adult	[18,33,58,98,102,108,118]
Blattodea	*Hodotermes mossambicus* (Hagen)	Harvester termite	Botswana	Larvae	[78]
Blattodea	*Macrotermes sp.*	Termite	Nigeria, Uganda	Adult queen, soldiers	[35,38,91]
Blattodea	*Syntermes soldiers*	Termite	Uganda	Adult	[38]
Blattodea	*Pseudacanthotermes militaris*	Termite	Kenya, Uganda	Winged adult	[59,108,115]
Blattodea	*Pseudacanthotermes spiniger*	Termite	Kenya, Uganda, Burundi	Winged adult	[59,108,115]
Blattodea	*Odontotermes kibarensis*	Termite	Uganda	Winged adult	[108]
Blattodea	*Pseudacanthotermes* sp.1	Termite	Uganda	Winged adult	[108]
Blattodea	*Pseudacanthotermes* sp.2	Termite	Uganda	Winged adult	[108]
Blattodea	*Odontotermes* spp.	Termite	Uganda	Winged adult	[108]
Blattodea	*Pseudacanthotermes* sp.5	Termite	Uganda	Winged adult	[108]
Blattodea	*Pseudacanthotermes* sp. 4	Termite	Burundi	Adult	[108]
Blattodea	*Macrotermes* spp.	Termite	Rwanda, Cameroon	Winged adult	[93,107,108]
Blattodea	*Macrotermes swaziae*	Termite	Zimbabwe		[33]
Blattodea	*Microhodotermes viator*	Termite	South Africa		[33]
Blattodea	*Termes badius*	Termite	South Africa	Winged adult	[33]
Lepidoptera	*Anaphe venata*	African silkworm	Nigeria, Zambia, Cote D’ivioire, Sierra Leona, Guinea, Liberia, Guinea Bissau, Angola	Larvae	[18,24,33,36,77,96]
Lepidoptera	*Anaphe infracta*	African silkworm	Nigeria	Larvae	[24,33]
Lepidoptera	*Anaphe recticulata*	African silkworm	Nigeria	Larvae	[24,33,36]
Lepidoptera	*Bunaea alcinoe*	Emperor moth	Nigeria; Democratic Republic of Congo, Botswana, Zimbabwe, Cameroon, Zambia, South Africa, Democratic Republic of Congo, Tanzania	Larvae, pupa and adult	[18,24,36,58,77,78,79,87,93,99,117]
Lepidoptera	*Lepidoptara litoralia*	Caterpillar	Nigeria	Larvae	[24,36,119]
Lepidoptera	*Cirina forda*	Pallid emperor	Nigeria, Angola, Democratic Republic of Congo, Botswana, Zimbabwe; Togo, Zambia, Mozambique, Ghana, Namibia	Larvae	[18,24,30,31,33,36,48,56,58,78,96,112,117,120,121,122,123]
Lepidoptera	*Imbrasia epimethea*	Caterpillar	Angola, Democratic Republic of Congo	Larvae	[18,33,45]
Lepidoptera	*Imbrasia obscura*	Caterpillar	Angola	Larvae	[96]
Lepidoptera	*Imbrasia truncata*	Caterpillar	Angola	Larvae	[96]
Lepidoptera	*Gonimbrasia* (*Nudaurelia*) *alopia*	Caterpillar	Angola	Larvae	[96]
Lepidoptera	*Gonimbrasia* (*Nudaurelia*) *dione*	Caterpillar	Angola	Larvae	[96]
Lepidoptera	*Pseudantheraea discrepans*	Caterpillar	Angola	Larvae	[96]
Lepidoptera	*Micragone cana*	Caterpillar	Angola, Democratic Republic of Congo	Larvae	[33,96]
Lepidoptera	*Anaphe panda*	Bagnest moth	Angola, Zimbabwe, Zambia, Cameroon, Democratic Republic of Congo, Nigeria, Tanzania	Larvae	[18,33,47,58,124]
Lepidoptera	*Notodontidae* sp. 1	Caterpillar	Angola	Larvae	[96]
Lepidoptera	*Notodontidae* sp. 2	Caterpillar	Angola	Larvae	[96]
Lepidoptera	*Notodontidae* sp. 3	Caterpillar	Angola	Larvae	[96]
Lepidoptera	*Notodontidae* sp. 4	Caterpillar	Angola	Larvae	[96]
Lepidoptera	*Gastroplakaeis rubroanalis*	Caterpillar	Angola	Larvae	[96]
Lepidoptera	*Sciatta inconcisa*	Caterpillar	Angola	Larvae	[96]
Lepidoptera	*Elaphrodes lactea* Gaede	Caterpillar	Democratic Republic of Congo	Larvae	[33,117]
Lepidoptera	*Lobobunaea saturnus* Fabricius	Caterpillar	Democratic Republic of Congo, Zimbabwe	Larvae	[33,58,117]
Lepidoptera	*Cinabra hyperbius* (Westwood)	Caterpillar	Democratic Republic of Congo	Larvae	[33,117]
Lepidoptera	*Gonimbrasia richelmanni* Weymer	Caterpillar	Democratic Republic of Congo	Larvae	[33,117]
Lepidoptera	*Antheua insignata*	Caterpillar	Democratic Republic of Congo	Larvae	[33,117]
Lepidoptera	*Imbrasia rubra*	Caterpillar	Democratic Republic of Congo	Larvae	[117]
Lepidoptera	*Athletes semialba* (Sonthonnax)	Caterpillar	Democratic Republic of Congo, Zimbabwe, Zambia, South Africa, Namibia, Mozambique	Larvae	[33,58,117]
Lepidoptera	*Cirina butyrospermi*	Caterpillar	Burkina Faso, Cote D’ivioire, Zambia, Zimbabwe, South Africa, Nigeria, Mali, Ghana	Larvae	[18,46,102,103,118,125]
Lepidoptera	*Hemijana variegata*	Caterpillar	South Africa	Larvae	[49]
Lepidoptera	*Imbrasia belina*	Mopane worm	Nigeria; Botswana; Zimbabwe, Namibia, South Africa, Malawi, Zambia, Angola, Mozambique	Larvae	[18,33,34,47,58,76,78,91,109,126,127,128,129]
Lepidoptera	*Isoberlina paniculata*	Caterpillar	Zambia	Larvae	[127]
Lepidoptera	*Urota sinope*	Caterpillar	Zambia, Botswana	Larvae	[78,130]
Lepidoptera	*Gonimbrasia zambesina*	Caterpillar	Zambia; Zimbabwe, Democratic Republic of Congo	Larvae	[33,58,130]
Lepidoptera	*Lophostethus dumolinii* Angas	Arrow sphinx	Botswana	Larvae	[78]
Lepidoptera	*Daphnis nerii L*	Oleander hawk moth	Botswana	Larvae	[78]
Lepidoptera	*Heniocha* spp.	Marbled emperor moth	Botswana	Larvae	[78]
Lepidoptera	*Imbrasia tyrrhea*	Willow emperor moth	Botswana	Larvae	[78]
Lepidoptera	*Sphingomorpha chlorea*	Sundown emperor moth	Botswana	Larvae	[78]
Lepidoptera	*Hippotion celerio* L.	Silver striped hawk	Botswana	Adult	[78]
Lepidoptera	*Agrius convolvuli* L.	Convolvulus hawk moth.	Botswana, South Africa, Angola, Zimbabwe, Zambia, Malawi, Mozambique, Namibia	Larvae	[78]
Lepidoptera	*Gonanisa maia*	Caterpillar	Zimbabwe, Botswana, Malawi, Democratic Republic of Congo, South Africa	Larvae	[33,47,58]
Lepidoptera	*Anthoaera zambezina*	Caterpillar	Zimbabwe, Botswana, Malawi, Namibia, Zambia, South Africa, Mozambique, Angola	Larvae	[33,58]
Lepidoptera	*Athletes gigas*	Caterpillar	Zimbabwe, Botswana, Malawi, Namibia, Zambia, South Africa, Mozambique, Angola	Larvae	[33,58]
Lepidoptera	*Bombycomorpha pallida*	Moth	Zimbabwe, South Africa	Larvae	[33,58]
Lepidoptera	*Bunaea caffra*	Moth	Zimbabwe, Zambia, South Africa, Namibia, Botswana, Mozambique, Angola	Larvae	[33,58]
Lepidoptera	*Bunaeopsis aurantica*	Moth	Zimbabwe, Democratic Republic of Congo	Larvae	[33,58]
Lepidoptera	*Gonometa postica*	Moth	Zimbabwe, South Africa	Larvae	[33,58]
Lepidoptera	*Heniocha dyops*	Moth	Zimbabwe, South Africa, Botswana, Zambia, Malawi, Namibia, Mozambique, Angola	Larvae	[33,58]
Lepidoptera	*Imbrasia epimethea*	Moth	Zimbabwe, Democratic Republic of Congo	Larvae	[33,58]
Lepidoptera	*Imbrasia ertli*	Caterpillar	Zimbabwe, South Africa, Cameroon, Democratic Republic of Congo, Angola, Zimbabwe, Botswana, Angola	Larvae	[18,33,58]
Lepidoptera	*Nudaurelia belina*	Moth	Zimbabwe, Malawi, Botswana, Mozambique, Namibia, Zambia, South Africa	Larvae	[33,58]
Lepidoptera	*Pseudobunaea irius*	Moth	Zimbabwe, South Africa, Zambia, Angola, Malawi, Namibia,	Larvae	[33,58]
Lepidoptera	*Loba leopardina*	Moth	Zimbabwe	Larvae	[58]
Lepidoptera	*Imbrasia oyemensis*	Caterpillar	Cote D’ivioire	Adult	[102]
Lepidoptera	*Imbrasia* spp.	Caterpillar	Cameroon	Larvae	[93]
Lepidoptera	*Eumeta* spp.	Caterpillar	Cameroon	Larvae	[107]
Lepidoptera	*Anaphe* spp.	Caterpillar	Cameroon	Larvae	[107]
Lepidoptera	*Dactyloceras* spp.	Caterpillar	Cameroon	Larvae	[107]
Lepidoptera	*Bunaea* spp.	Caterpillar	Cameroon	Larvae	[107]
Lepidoptera	*Dactyloceras lucina*	Caterpillar	Democratic Republic of Congo, Zambia, South Africa, Cameroon, Angola, Gabon, Sierra Leone, Equatorial Guinea, Sao Tome,	Larvae	[18]
Lepidoptera	*Platysphinx stigmatica*	Caterpillar	Zambia, Democratic Republic of Congo, Sierra Leone, Rwanda, Burundi, Equatorial Guinea, Sao Tome,	Larvae	[18]
Lepidoptera	*Epanaphe carteri*	Caterpillar	Democratic Republic of Congo, Angola, Gabon, Sierra Leone, Sao Tome, Equatorial Guinea	Larvae	[18]
Lepidoptera	*Gynanisa ata*	Caterpillar	Democratic Republic of Congo, Zambia, Malawi, Sudan	Larvae	[18]
Lepidoptera	*Eumeta cervina*	Caterpillar	Democratic Republic of Congo, Cameroon, Angola, Gabon, Sierra Leone, Sao Tome, Equatorial Guinea, Rwanda, Burundi, Liberia	Larvae	[18]
Lepidoptera	*Urota sinope*	Caterpillar	Democratic Republic of Congo, South Africa, Zimbabwe, Zimbabwe, Botswana, Gabon, Mozambique, Namibia	Larvae	[18,33]
Lepidoptera	*Anthoaera caffraria*	Caterpillar	Angola, Malawi, South Africa, Zambia, Zimbabwe, Mozambique, Namibia, Botswana	Larvae	[33]
Lepidoptera	*Anthoaera menippe*	Caterpillar	Angola, Malawi, South Africa, Zambia, Zimbabwe, Mozambique, Namibia, Botswana	Larvae	[33]
Lepidoptera	*Bunaea caffraria*	Caterpillar	Democratic Republic of Congo	Larvae	[33]
Lepidoptera	*Drapetides uniformis*	Caterpillar	Democratic Republic of Congo	Larvae	[33]
Lepidoptera	*Gonimbrasia hecate*	Caterpillar	Democratic Republic of Congo	Larvae	[33]
Lepidoptera	*Goodia kuntzei*	Caterpillar	Democratic Republic of Congo	Larvae	[33]
Lepidoptera	*Heniocha apollonia*	Caterpillar	Angola, Malawi, South Africa, Zambia, Zimbabwe, Mozambique, Namibia, Botswana	Larvae	[33]
Lepidoptera	*Heniocha marnois*	Caterpillar	Angola, Malawi, South Africa, Zambia, Zimbabwe, Mozambique, Namibia, Botswana	Larvae	[33]
Lepidoptera	*Herse convolvuli*	Caterpillar	South Africa	Larvae	[33]
Lepidoptera	*Imbrasia dione*	Caterpillar	Democratic Republic of Congo	Larvae	[33]
Lepidoptera	*Imbrasia macrothyris*	Caterpillar	Democratic Republic of Congo	Larvae	[33]
Lepidoptera	*Imbrasia rubra*	Caterpillar	Democratic Republic of Congo	Larvae	[33]
Lepidoptera	*Lobobunaea phaedusa*	Caterpillar	Democratic Republic of Congo	Larvae	[33]
Lepidoptera	*Melanocera parva*	Caterpillar	Democratic Republic of Congo	Larvae	[33]
Lepidoptera	*Microgene cana*	Caterpillar	Democratic Republic of Congo	Larvae	[33]
Lepidoptera	*Nudaurelia macrothyrus*	Caterpillar	Democratic Republic of Congo	Larvae	[33]
Lepidoptera	*Nyodes prasinodes*	Caterpillar	Democratic Republic of Congo	Larvae	[33]
Lepidoptera	*Rohaniella pygmaea*	Caterpillar	Angola, Malawi, South Africa, Zambia, Zimbabwe, Mozambique, Namibia, Botswana	Larvae	[33]
Lepidoptera	*Rheneae mediata*	Caterpillar	Democratic Republic of Congo	Larvae	[33]
Lepidoptera	*Tagoropsis flavinata*	Caterpillar	Democratic Republic of Congo	Larvae	[33]
Lepidoptera	*Usta terpisichore*	Caterpillar	Angola	Larvae	[33]
Lepidoptera	*Usta wallengreni*	Caterpillar	Angola, Malawi, South Africa, Zambia, Zimbabwe, Mozambique, Namibia, Botswana	Larvae	[33]
Mantodea	*Mantis religiosa*	African mantis	Nigeria, South Africa	Adult	[33,79]
Orthoptera	*Brachytrupes membranaceus*	Giant African cricket	Nigeria, Angola; Zimbabwe, Uganda; Cameroon, Democratic Republic of Congo, Burkina Faso, Tanzania, Angola, Togo, Benin; Malawi	Adult	[18,24,33,36,45,53,58,77,79,91,93,96,99,108,124]
Orthoptera	*Gymnogryllus lucens*	Cricket	Nigeria	Adult	[24,36,116]
Orthoptera	*Cytacanthacris naeruginosus*	Short horned grasshopper	Nigeria	Adult	[24,36]
Orthoptera	*Zonocerus variegatus*	Grasshopper	Nigeria, Cameroon, Uganda, Democratic Republic of Congo, Cote D’ivioire, Ghana, Guinea, Liberia, Sao Tome, Liberia, Guinea Bissau	Adult	[18,24,35,36,38,77,79,85,94,99,110,116,131]
Orthoptera	*Gryllotalpa africana*	Mole cricket	Nigeria; Zimbabwe; Malawi	Adult	[24,33,36,47,77,79,99,124]
Orthoptera	*Ruspolia differens*	Grasshopper	Kenya, Tanzania, Democratic Republic of Congo, Uganda, Zimbabwe, Rwanda, Cameroon, Uganda, Malawi, South Africa	Adult	[33,58,59,60,108,112,115,117,132]
Orthoptera	*Melanoplus foedus*	Grasshopper	Nigeria	Adult	[112]
Orthoptera	*Gryllus assimilis*	Cricket	Nigeria; Ghana	Adult	[94,112]
Orthoptera	*Henicus whellani*	Cricket	Zimbabwe	Adult	[50,51]
Orthoptera	*Kraussaria ongulifera*	Grasshopper	Burkina Faso	Adult	[98,118]
Orthoptera	*Gryllus campestris*	Field cricket	Burkina Faso; Cameroon, Malawi	Adult	[98,107,124]
Orthoptera	*Ruspolia nitidula*	Grasshopper	Uganda	Larvae and adult	[22,133]
Orthoptera	*Normadacris septemfasciata*	Red locust	Botswana; Uganda, Zambia, South Africa, Democratic Republic of Congo, Zimbabwe, Botswana, Nigeria, Uganda, Tanzania, Malawi, Mozambique	Adult	[18,78,108]
Orthoptera	*Locustana pardalina*	Brown locust	Botswana, South Africa, Zimbabwe, Malawi, Libya	Adult	[18,33,78]
Orthoptera	*Schistocerca gregaria*	Desert locust	Botswana, Zambia, South Africa, Cameroon, Democratic Republic of Congo, Zimbabwe, Burkina, Faso, Malawi, Mali, Niger, Togo, Benin	Adult	[18,78,116]
Orthoptera	*Cyrtacanthacris tatarica* L	Brown-spotted locust	Botswana	Adult	[78]
Orthoptera	*Acrida acuminata*	Common stick grasshopper	Botswana	Adult	[78]
Orthoptera	*Zonocerus elegans*	Elegant grasshopper	Botswana, South Africa	Adult	[33,78]
Orthoptera	*Acrotylus* spp.	Burrowing grasshopper	Botswana	Adult	[78]
Orthoptera	*Homorocoryphus nitidulus*	Cricket	Cameroon	Larvae	[85,107]
Orthoptera	*Gynanisa maia*	Cricket	Zimbabwe, Malawi, South Africa	Larvae	[33,58]
Orthoptera	*Locusta migratoria*	migratory locust	Zimbabwe, Cote D’ivioire; Nigeria; Sudan, Zambia, Democratic Republic of Congo, Sudan, Ghana	Adult	[18,33,79,94,102,134,135]
Orthoptera	*Acheta domesticus*	Cricket	Cote D’ivioire; Nigeria, Ghana	Adult	[79,94,102]
Orthoptera	*Cartarrtopsilus taeniolatus*	Grasshopper	Nigeria	Adult	[35]
Orthoptera	*Zulua cyanoptera*	Grasshopper	Nigeria	Adult	[35]
Orthoptera	*Brachytrupes spp.*	Cricket	Uganda, Cameroon	Adult	[107,110]
Orthoptera	*Cyrtacanthacris aeruginosa* unicolor	Grasshopper	Uganda	Adult	[110]
Orthoptera	*Zonocerus sp.*	Grasshopper	Nigeria	Adult	[91]
Orthoptera	*Daraba* (Sceloides) *laisalis*	Locust	Nigeria	Larvae, pupa, adult	[91]
Orthoptera	*Ornithacris turbida*	Grasshopper	Zimbabwe	Adult	[47]
Orthoptera	*Acanthoplus discoidalis*	Cricket	Zimbabwe	Adult	[47]
Orthoptera	*Acanthacris ruficornis*	Garden locust	Uganda, Zambia, South Africa, Cameroon, Democratic Republic of Congo, Zimbabwe, Burkina Faso, Malawi, Mali, Niger, Togo, Benin	Adult	[18,108]
Orthoptera	*Schistocerca* spp.	Grasshopper	Cameroon	Adult	[107]
Orthoptera	*Acanthacris* spp.	Grasshopper	Cameroon	Adult	[107]
Orthoptera	*Gastrimargus* spp.	Locust	Cameroon	Adult	[107]
Orthoptera	*Phymateus* spp.	Locust	Cameroon	Adult	[107]
Orthoptera	*Anacridium* spp.	Locust	Cameroon	Adult	[107]
Orthoptera	*Pyrgomorpha* spp.	Locust	Cameroon	Adult	[107]
Orthoptera	*Gastrimargus africanus*	Locust	Cameroon, Democratic Republic of Congo, Niger, Lesotho, Liberia	Adult	[18]
Orthoptera	*Phymateus viridipes brunneri* Bolivar	Locust	Zambia, South Africa, Democratic Republic of Congo, Zimbabwe, Botswana, Mozambique, Namibia	Adult	[18]
Orthoptera	*Gryllus bimaculatus*	Cricket	Togo, Nigeria, Guinea Bissau, Sierra Leone, Liberia, Benin, Democratic Republic of Congo, Kenya, Sudan, Zambia	Adult	[18]
Orthoptera	*Anacridium melanorhodon melanorhodon*	Cricket	Cameroon, Sudan, Niger	Adult	[18,52]
Orthoptera	*Paracinema tricolor*	Cricket	Cameroon, Malawi, Lesotho	Adult	[18]
Orthoptera	*Acheta* spp.	Cricket	Zambia, Zimbabwe, Kenya	Adult	[18]
Orthoptera	*Scapteriscus vicinus*	Field cricket	Ghana	Adult	[94,103]
Orthoptera	*Gryllotalpa gryllotalpa*	Mole cricket	Malawi	Adult	[124]
Orthoptera	*Homorocoryphus vicinus*	Cricket	Uganda	Adult	[33]
Orthoptera	*Nomadacris septumfasciata*	Cricket	South Africa	Adult	[33]
Orthoptera	*Schistocerca gregaria*	Cricket	Zimbabwe	Adult	[33]
Blattodea	*Pseudacathotermes spinige*	Termite	Kenya	Adult	[37]
Blattodea	*Macrotermes* spp.	Termite	Kenya	Adult	[37]
Blattodea	*Macrotermes subhylanus*	Termite	Kenya	Adult	[37]
Hymenoptera	*Crematogaster mimosae*	Ant	Kenya	Adult	[37]
